# High tumor CD161 expression predicts a survival advantage and marks a Th1-skewed microenvironment

**DOI:** 10.3389/fimmu.2025.1522755

**Published:** 2025-03-17

**Authors:** Briana Amicarella Burns, Manasvi Chandra, Vanaja Konduri, William K. Decker

**Affiliations:** ^1^ Department of Pathology & Immunology, Baylor College of Medicine, Houston, TX, United States; ^2^ Dan L. Duncan Cancer Center, Baylor College of Medicine, Houston, TX, United States; ^3^ Center for Cell and Gene Therapy, Baylor College of Medicine, Houston, TX, United States

**Keywords:** cancer, immune response, dendritic cell, T-cell, CD161

## Abstract

CD8^+^CD161^+^ T-cells exhibit augmented memory and cytolytic properties, mediating enhanced immunity in murine tumor models and improved survival in human non-small cell lung cancer. This T-cell subset might serve as a biomarker of positive response to therapy or even be isolated to augment current immunotherapeutic approaches yet limited knowledge of CD161 expression in human cancers restricts practical application. Here we bioinformatically tested the hypothesis that CD161 expression may be associated with positive outcomes in human cancers and investigated mechanisms underlying any observed advantages. Using TCGA-PANCAN dataset, we analyzed expression of CD161 in over 10,000 human tumors, correlating expression levels with survival. CD161 expression was highly correlated and largely co-expressed with CD8, indicating that observed benefits could be attributed to CD8^+^CD161^+^ T-cells. While patients with high CD161 expression exhibited a clear survival advantage over those with low expression, this survival advantage was highly dependent on co-expression of CD11c, indicating a reliance on dendritic cells (DC). To further explore the mechanism by which high CD161 expression confers a survival advantage in cancer, we analyzed available scRNA-sequencing data derived from 31 melanoma tumors. Tumors exhibiting high CD8^+^CD161^+^ infiltration also exhibited greater expression of cDC1 and T_H_1 transcription factors along with higher levels of inflammatory cytokine transcripts. CD8^+^CD161^+^ cells themselves displayed enhanced cytotoxicity markers and reduced exhaustion markers compared to CD8^+^CD161^neg^ T-cells. The data suggest that CD161 could serve as a biomarker for positive outcomes and that DC play a critical *in vivo* role in the propagation of CD161^+^ T-cell responses.

## Introduction

CD8^+^CD161^+^ T-cells have garnered significant interest due to properties associated with enhanced cytotoxicity, reduced exhaustion, and durable immunologic memory (1–12). CD161, also known as NK1.1 in mice, was first identified as a marker of natural killer (NK) cell innate cytotoxicity but is now known to also be expressed on both CD8 and CD4 T-cells with diverse ab-TCR repertoires, suggesting a role beyond that associated with natural cytotoxicity (8, 13, 14). CD161^+^ T-cells have demonstrated a potent ability to mediate long-lasting immunity, particularly in a murine model of pancreatic ductal adenocarcinoma (PDAC) in which adoptively transferred CD8^+^NK1.1^+^ cells provided durable protection and improved survival with similar results shown when CD8^+^CD161^+^ T-cells were isolated for use in CAR T-cell therapy (2, 15). Gene expression analyses have further highlighted elevated levels of granzyme, perforin, other innate-like receptors, and a chemokine receptor profile indicative of tissue extravasation, underscoring a potential role for such cells in durable antitumor immunity (2).

Despite compelling evidence from murine models, the general relevance of CD161^+^ T-cells to human tumor immunity remains unclear. While CD161 expression is limited to human lymphocyte populations, the effect on these populations is complicated, with high level expression noted on TCR Va7.2 invariant MR1-restricted MAIT cells (16, 17), induced expression on most T-cell subsets upon activation, and the functional identification of CD161 as an inhibitory receptor on NK cells and some T-cells (18). However, while MAIT cells may be found in circulation and at mucosal surfaces such as the lamina propria, these cells are not known to extravasate into tissue spaces or tumors. Moreover, identification of CD161 as an inhibitory receptor on NK cells and some T-cells does not preclude the possibility that its specific expression on T-cells marks a highly cytolytic memory effector cell as suggested by the literature (2), but simply that such characteristics may be subject to an additional level of regulation. In specific human cancers, CD161 has been implicated in immune evasion, immune cell infiltration, and survival outcomes, highlighting a potentially complex role in the tumor microenvironment (18–22). While CD161^+^ T-cells confer a survival advantage in multiple murine tumor models (2), the survival advantage of this T-cell subset remains unknown. Furthermore, while prior studies suggest that dendritic cells (DCs) can promote the expansion and function of CD161^+^ T-cells, the factors regulating their presence in human tumor have yet to be fully elucidated. A deeper understanding of CD161^+^ cells in the specific context of cancer is necessary to determine their therapeutic potential.

In this study, we sought to determine whether the findings from murine models, in which CD8^+^CD161^+^ T-cells play a protective role in tumor immunity, are translatable to human cancers. Using a bioinformatic approach, we tested the hypothesis that these cytolytic memory effector cells offer a survival advantage in human tumors. Additionally, we explored factors that might shape their presence and function in the tumor microenvironment to better understand whether similar mechanisms to those observed in murine models drive their function in human tumors. Understanding such elements could provide insights into the use of CD8^+^CD161^+^ cells in active immunotherapeutic approaches as well as broader treatment implications.

## Methods

### TCGA data analysis

Survival and gene expression data for this study were obtained from The Cancer Genome Atlas (TCGA) pan-cancer (PANCAN) data set through UCSC Xena database. The gene expression data from the database is measured in transcripts per million (TPM). For each gene of interest (listed in **Supplementary Table 1**), the expression level was divided into tertiles, categorized as low, medium, and high expression groups as described in previous studies (23–25). In subsequent analyses, we compare the survival outcome of patients in the low tertile to those in the high tertile to identify significant associations that may be stratified by gene expression.

### scRNA sequencing data analysis

For single-cell RNA sequencing (scRNA-seq) analysis, we used data from the Gene Expression Omnibus (GEO) database that included scRNA-seq profiles from 31 melanoma patients (GEO accession: GSE115978) (26). Previously defined cell types were used via GEO annotations. Immune cells were defined by filtering out cancer-associated fibroblasts, endothelial cells, and malignant cells. Myeloid cells were defined by filtering out NK cells, T-cell, and B-cells from the immune cell subset. To define CD8^+^CD161^+^ infiltration, we categorized patients into low or high infiltration based on their rank in the distribution of the percentage of CD8^+^CD161^+^ cells relative to the total cell count (range = 0%-11.6%, median = 2.0%). For each gene of interest (listed in **Supplementary Tables 1**, **2**), we compared both the average expression of the gene and the percentage of cells expressing that gene in tumors with low or high CD8^+^CD161^+^ infiltration. T-cell functional markers (listed in **Supplementary Tables 1**, **2**) were compared between CD8^+^CD161^+^ and CD8^+^CD161^neg^ cells.

### Statistical analysis

All statistical analyses were performed in R version 4.4.0. Pearson correlation (*stat_cor* {*ggpubr*}) was used to assess the linear correlation between variables. Chi-squared test (*chisq.test* {*stats*}) was used to analyze associations within 2x2 contingency tables. Survival analysis was conducted using the log-rank test (*survdiff* {*survival*}). Cox proportional regression was used to compare survival advantages between groups (*coxph* {*survival*}). When comparing two groups, Student’s t-test (*t.test* {*stats*}) was applied to normally distributed data and Wilcoxon rank-sum test (*wilcox.test* {*stats*}) was used for non-normal data. All plots were generated using *ggplot2.* Statistical significance was defined as p ≤ 0.05.

## Results

### CD161 marks a survival advantage in human tumors

**Figure 1:**
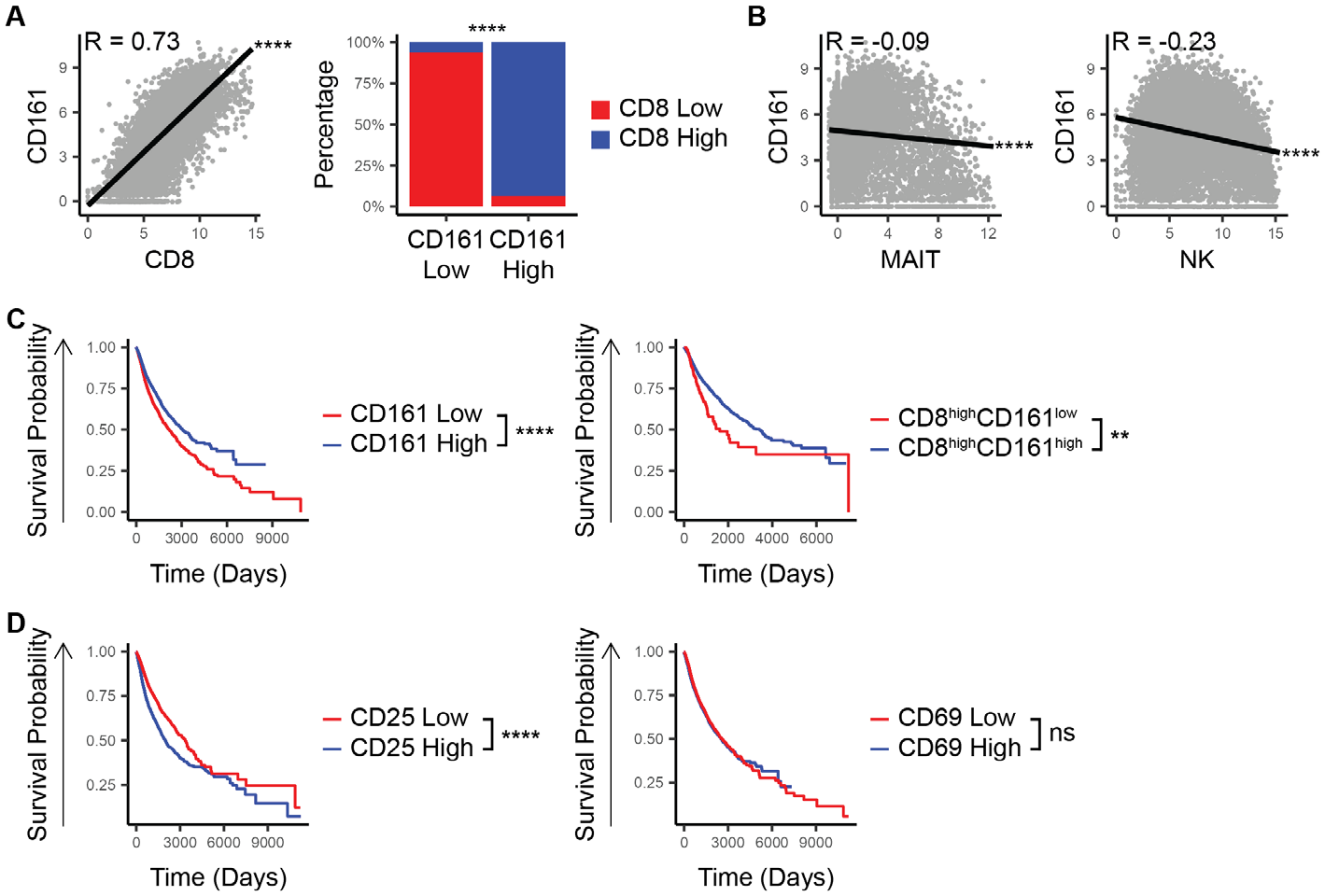
CD8 and CD161 co-expression marks a unique survival advantage in TCGA patients. **(A)** Correlation between CD161 and CD8 and proportion of CD8 low vs high within the CD161 low vs high groups. **(B)** Correlation between the expression of CD161 and MAIT cell marker. **(C)** Survival based on the expression of CD161 and CD8. **(D)** Survival based on the expression of CD161 and CD25 or CD69. ns (not significant), ** *p < 0.01*, **** *p < 1E-4*.

In an analysis of the TCGA-PANCAN database, we observed a strong positive correlation between CD8 and CD161 expression (R = 0.73, p < 10^-15^, **Figure 1A**) with CD8 being largely co-expressed with CD161 (p < 10^-15^, **Figure 1A**). This co-expression pattern suggests that the potential survival advantage associated with CD161 is likely attributable to its presence in CD8^+^ T-cells rather than being an independent effect. To assess whether CD161 expression is influenced by other immune cell populations, we examined the correlation between CD161 and markers of MAIT (SLC4A10) and NK (CD56/NCAM1) cells. Both showed negative correlations with CD161 expression (p < 10^-15^, **Figure 1B**), indicating that the observed CD161 is unlikely to be derived from these cell types.

When examining patient outcomes, those with high CD161 expression consistently demonstrated better overall survival compared to those with lower CD161 levels (p = 7.4E-12, **Figure 1C**). This trend held true even within the subset of patients who already had high CD8 expression (p = 0.002, **Figure 1C**). To determine whether CD161 is merely a marker of overall immune activation rather than a direct contributor of survival, we analyzed the survival association of CD25, a marker of T-cell activation, and CD69, a general immune activation marker. Unlike CD161, high CD25 expression was associated with a negative survival outcome, while CD69 expression showed no significant difference in survival (p < 10^-15^, p = 0.3, **Figure 1D**), suggesting that CD161’s role extends beyond general immune activation. These findings underscore the pivotal role of CD8^+^CD161^+^ cells in driving a more effective anti-tumor immune response, ultimately contributing to improved patient outcomes.

### The CD161 survival advantage is dependent on dendritic cells and further enhanced by the presence of DCs

**Figure 2:**
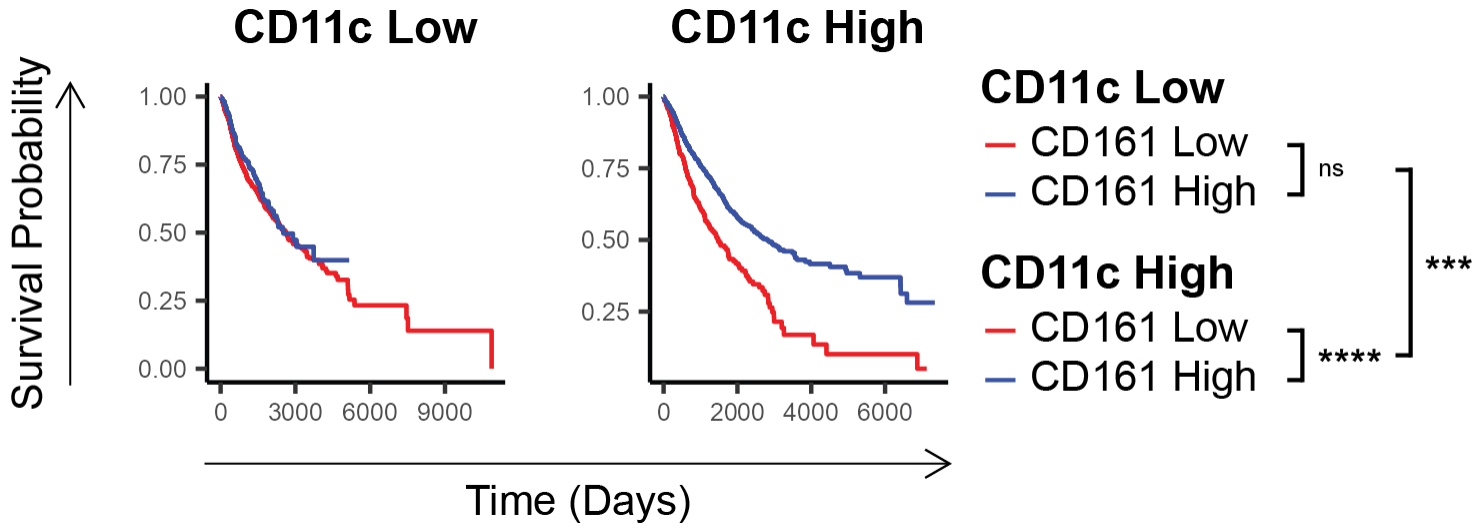
CD161 survival advantage is dependent on the expression of cDC1 markers. Comparison of survival of individuals in TCGA having low vs high CD161 expression in those who express low vs high CD11c. ns (not significant), *** p < 0.001, **** p < 1E-4.

To further explore the mechanisms underlying the observed survival advantage correlated with CD161 expression, we conducted a comprehensive analysis of various immune drivers [detailed in **Supplementary Table 1**; (15, 24–28)]. This analysis revealed that the survival benefit of CD161 was wholly contingent upon CD11c expression, highlighting a potential dependency on dendritic cells (p_CD11c-low_ = 0.2, p_CD11c-high_ = 1E-12, p_interaction_ = 1E-5, **Figure 2**).

### CD161 cells promote a Th1 skewed environment in melanoma

**Figure 3:**
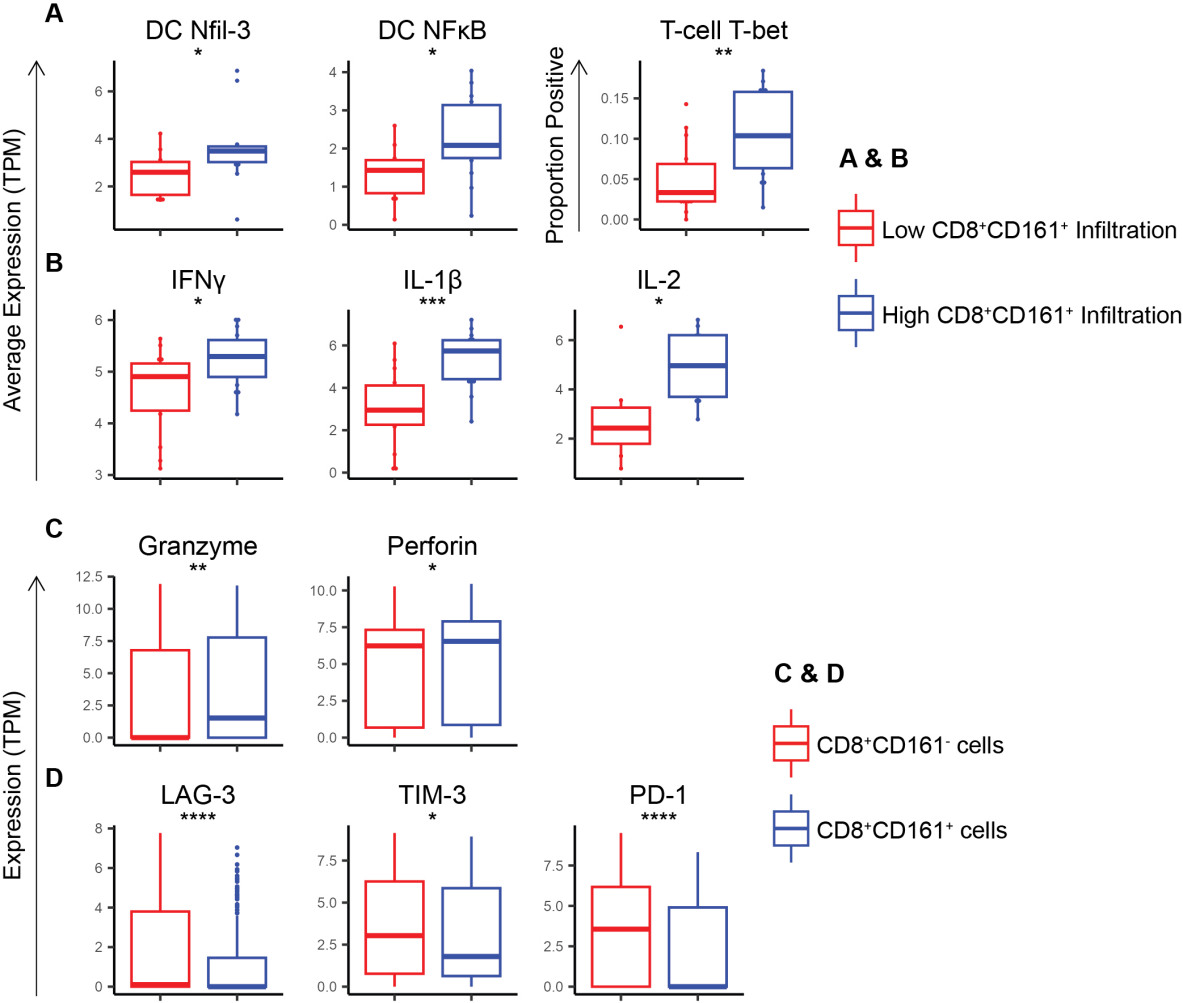
scRNA sequencing of melanomas show that co-expression of CD8 and CD161 skews the immune response towards a Th1 response while enhancing cytotoxicity markers and inhibiting exhaustion markers in CD8 T-cells. **(A)** Average expression of Th-1 skewing transcription factors within DCs and T-cells in patients with high vs low CD8^+^CD161^+^ T-cell infiltration. **(B)** Average expression of Th-1-associated cytokines within immune cells in patients with high vs low CD8^+^CD161^+^ T-cell infiltration. **(C)** Expression of cytotoxicity markers within CD8^+^CD161^+^ vs CD8^+^CD161^neg^ cells. **(D)** Expression of exhaustion markers within CD8^+^CD161^+^ vs CD8^+^CD161^neg^ cells. * p < 0.05, ** p < 0.01, *** p < 0.001, **** p < 1E-4..

In further investigation of the cellular mechanisms underlying the observed survival advantage conferred by CD8^+^CD161^+^ cells, we analyzed high quality single-cell RNA sequencing (scRNA-seq) data derived from 31 melanoma tumors. This analysis focused on a broad range of immune markers [listed in **Supplementary Tables 1**, **2**; (15, 24–29)] within specific cell populations. We categorized patients into groups based on the level of CD8^+^CD161^+^ cell infiltration, defining high and low infiltration based on the percentage of total cells that were CD8^+^CD161^+^. Patients with high infiltration of CD8^+^CD161^+^ T-cells exhibited substantially higher expression levels of Nfil-3 (p = 0.05), a critical transcription factor in the differentiation of cDC1s, and NFκB (p = 0.02, a transcription factor responsible for production of IL-12 and subsequent T_H_1 responses, in their myeloid cell populations (**Figure 3A**). Additionally, a higher proportion of T-cells express T-bet (p = 0.003) (**Figure 3A**) marking a T_H_1 response. Taken together, these data suggest that cDC1s could be mediating a T_H_1-polarizing immune response in these patients (27). Moreover, patients with a high percentage of infiltrating CD8^+^CD161^+^ T-cells also showed elevated levels of inflammatory cytokines, including IFNγ (p = 0.02), IL-1β (p = 5E-4), and IL-2 (p = 0.01), often associated with T_H_1 responses (**Figure 3B**). When examining the CD8^+^CD161^+^ cells as a group, these cells were found to express higher levels of cytotoxicity markers, including granzyme (p = 0.001) and perforin (p = 0.02), in comparison to CD8^+^CD161^neg^ cells (**Figure 3C**). Additionally, CD8^+^CD161^+^ T-cells demonstrated lower expression of exhaustion markers, LAG-3 (p = 3E-7), TIM-3 (p = 0.05), and PD-1 (p = 3E-7), pointing towards a more robust and sustained anti-tumor response (**Figure 3D**). Taken together, these findings demonstrate that in a T_H_1-polarizing tumor microenvironment, CD8^+^CD161^+^ T-cells exhibit enhanced cytotoxicity and reduced exhaustion, potentially promoting survival in melanoma patients.

## Discussion

In this study, we tested the hypothesis that CD8^+^CD161^+^ T-cells confer a survival advantage in human cancers and focused on mechanistic underpinnings that might drive this effect. This study builds on our prior word demonstrating the functional capabilities of CD8^+^CD161^+^ T-cells, including their cytotoxicity and durability in murine models of PDAC and their potential in CAR-T-cell therapy (2). Our findings strongly suggest that CD8^+^CD161^+^ cells, particularly in conjunction with dendritic cells, play a critical role in enhancing anti-tumor immunity and improving patient outcomes.

The study revealed the CD8 and CD161 are highly correlated and co-expressed across various cancers, suggesting that the survival advantage associated with CD161 is largely due to these specific co-expressing T-cells. We found that CD161 expression is linked to significantly improved survival, even within the CD8-high subset, emphasizing a distinct functional role of this subset. Further, the survival advantage of CD161 appears to be dependent on dendritic cells. While the analysis of TCGA data indicated that the survival advantage associated with CD161 expression is consistent across a range of tumor types, we acknowledge the potential for inter-tumor heterogeneity to influence the immune microenvironment and the specific role of CD161. Future studies should investigate the tumor-specific mechanisms by which CD161 contributes to immune regulation and survival outcomes, considering the unique immune landscapes of different cancer types.

Our findings indicate that the survival advantage associated with CD161^+^ T-cells is linked to DCs, as demonstrated by the dependence on CD11c expression in TCGA data. While CD11c is not exclusive to cDC1, our scRNA-seq analysis shows upregulation of cDC1 differentiation and activation markers in tumors with high CD8^+^CD161^+^ T-cell infiltration, suggesting a potential role for cDC1 in driving CD161^+^ T-cell differentiation. Furthermore, our previous work demonstrated that an infusion of cDC1 skewed DCs leads to enrichment of CD8^+^CD161^+^ T-cells, further supporting the idea that cDC1 may play a role in their expansion or activation (2, 15, 28). Future studies will aim to determine mechanism by which cDC1s contribute to CD8^+^CD161^+^ T-cell function.

The results further demonstrate that CD8^+^CD161^+^ T-cells can comprise a crucial component of the anti-tumor immune response, conferring a significant survival advantage in human cancers. The Th1-skewed response and reduced exhaustion observed in CD8^+^CD161^+^ cells in conjunction with cDC1 skewing further underscore the importance of this subset. However, further studies are required to define the mechanisms by which CD161 influences the cytotoxicity and exhaustion pathways in T-cells.

While RNA-seq data provides valuable insights in gene expression levels, it is important to note that mRNA levels may not directly reflect protein abundance or functional activity. This limitation highlights the need for further experimental validation to confirm the biological relevance of CD8^+^CD161^+^ T-cells as indicated by their transcriptional profile.

These findings suggest that enhancing the function or proliferation of CD8^+^CD161^+^ T-cells or modulating the activity of cDC1s to favor the production of these T-cells, could be a promising strategy in cancer immunotherapy. Not only could the presence of CD8^+^CD161^+^ T-cells serve as a biomarker to predict response to immunotherapy, but these cells also represent a distinct cytotoxic subset that could be harnessed in multiple immunotherapeutic strategies. Their reduced exhaustion phenotype and strong Th1-skewed response make them an attractive candidate for adoptive cell transfer approaches, including CAR T-cell therapy, where strategies to expand and enrich this subset could improve therapeutic outcomes. Given our findings that cDC1s may contribute to the presence and function of these cells, DC vaccines or adjuvants that enhance cDC1 priming couple serve as a complementary strategy to increase CD8^+^CD161^+^ T-cell infiltration into tumors. Future studies should explore how these approaches can be integrated into current immunotherapy protocols to improve patient outcomes. These findings establish CD8^+^CD161^+^ T-cells as a potent immune subset with strong implications for cancer therapy. Their ability to confer a survival advantage, driven by their priming by cDC1s and their intrinsic functional properties, highlights their potential as a both a biomarker and a target for enhancing immunotherapeutic efficacy.

## Data Availability

The original contributions presented in the study are included in the article/**Supplementary Material**. Further inquiries can be directed to the corresponding author.
