# Supplementary material for: High tumor CD161 expression predicts a survival advantage and marks a Th1-skewed microenvironment
Source: Front Immunol. 2025 Mar 17;16:1522755. doi: 10.3389/fimmu.2025.1522755 (PMC11955640; doi:10.3389/fimmu.2025.1522755)
Supplement: Supplementary file 1 [file Table1.docx]

**Supplementary Table 1.** T-cell markers used in the analysis of both the TCGA and the scRNA sequencing data set.

| **Skew** | **Cell Type** | **Marker** | **Source** |
| --- | --- | --- | --- |
| Th1 | cDC1 | Xcr1 | (24, 25) |
|  |  | Batf3 | (24, 25) |
|  |  | Nfil3 | (24, 25) |
|  |  | NFκB | (26) |
|  | pDC | Tcf4 | (24, 25) |
|  | T-cell | T-bet | (15,27) |
|  |  | Stat-1 | (27) |
|  | Cytokines (all cells) | IL-12 | (27) |
|  |  | IL-2 | (27) |
|  |  | IFNγ | (27) |
|  |  | TNFα | (27) |
| Th2 | cDC2 | Cx3cr1 | (24, 25) |
|  |  | IRF4 | (24, 25) |
|  | T-cell | Gata-3 | (15,27) |
|  |  | Stat-5 | (15,27) |
| Th17 | T-cell | Rorc | (15,27) |
|  |  | Stat-3 | (15,27) |
| Regulatory | Cytokines (all cells) | IL-10 | (27) |
| Maturity | DC | CD80 | (28) |
|  |  | CD86 | (28) |
|  |  | CD11c | (25) |
|  |  | CD11b | (25) |
|  |  | SIRPα | (28) |
