# Supplementary material for: High tumor CD161 expression predicts a survival advantage and marks a Th1-skewed microenvironment
Source: Front Immunol. 2025 Mar 17;16:1522755. doi: 10.3389/fimmu.2025.1522755 (PMC11955640; doi:10.3389/fimmu.2025.1522755)
Supplement: Supplementary file 2 [file Table2.docx]

**Supplementary Table 2.** T-cells markers used in addition to the markers listed in Supplementary Table 1 in the analysis of the scRNA sequencing data set.

| **Skew** | **Cell Type** | **Marker** | **Source** |
| --- | --- | --- | --- |
| Th1 | pDC | Clec4c | (24, 25) |
|  | T-cell | Stat-4 | (15,27) |
| Th2 | Cytokines (all cells) | IL-4 | (27) |
|  |  | IL-5 | (27) |
|  |  | IL-13 | (27) |
| Th17 | Cytokines (all cells) | IL-6 | (27) |
|  |  | IL-1b | (27) |
|  |  | IL-17 | (27) |
|  |  | IL-23 | (27) |
| Regulatory | T-cell | TGFb | (27) |
|  |  | Foxp3 | (15,27) |
|  |  | LAG-3 | (29) |
|  |  | TIM-3 | (29) |
|  |  | PD-1 | (29) |
